# Frequency Shift of a SH-SAW Biosensor with Glutaraldehyde and 3-Aminopropyltriethoxysilane Functionalized Films for Detection of Epidermal Growth Factor

**DOI:** 10.3390/bios10080092

**Published:** 2020-08-05

**Authors:** Xue-Chang Lo, Jen-Yu Li, Ming-Tsang Lee, Da-Jeng Yao

**Affiliations:** 1 Department of Engineering and System Science, National Tsing Hua University, Hsinchu 30013, Taiwan; lo107011531@gapp.nthu.edu.tw (X.-C.L.); djyao@mx.nthu.edu.tw (D.-J.Y.); 2 Institute of NanoEngineering and MicroSystems, National Tsing Hua University, Hsinchu 30013, Taiwan; doraemon80508@hotmail.com; 3 Department of Power Mechanical Engineering, National Tsing Hua University, Hsinchu 30013, Taiwan

**Keywords:** SH-SAW biosensor, 3-aminopropyltriethoxysilane, glutaraldehyde, epidermal growth factor (EGF)

## Abstract

The frequency shift of a shear-horizontal surface-acoustic-wave (SH-SAW) biosensor in which the concentration of biomolecule is determined by the amount of its adsorption on the sensing film was studied. Simulation results were compared with experimental results to investigate its sensitivity and to develop a model to estimate the concentration of a cancer-related biomarker antigen epidermal growth factor (EGF) in the sample, with two types of sensing films, 3-aminopropyltriethoxysilane (APTES) and glutaraldehyde. With the concentration of the targeted biomarker varying from 0.2 to 5 ng/mL, a typical exponential relationship was found between the concentration and the frequency shift of the SH-SAW sensor. Measurement results showed a clear response of this immunosensor to the mass-loading effects of the antibody–antigen. The sensitivity of the glutaraldehyde film is greater than that of the APTES film owing to the chemisorption of the antibody. In the simulation, a shift of the SH-SAW resonant frequency due to added mass occurred on applying an incremental surface mass density on the sensing film, while in real applications, the concentration of the targeted biomarker to be absorbed in the sensing film is demanded. An empirical model was proposed to calculate the frequency shift in the simulation of the SH-SAW biosensor, corresponding to the concentration of specific biomolecules absorbed on a specific film. From the semi-empirical model, the sensitivity level is found to be 0.641 and 1.709 kHz/(ng/mL) for APTES and glutaraldehyde sensing films, respectively, at a biomarker concentration of less than 1 ng/mL. The developed method is useful for quickly estimating the frequency shift with respect to the concentration of the target molecules in the simulation for SH-SAW sensors.

## 1. Introduction

Surface-acoustic-wave (SAW) sensors, with their advantages of high sensitivity, low cost, low power requirement and real-time monitoring, have great potential in the application of immune sensing, environmental monitoring and biotechnology. Biosensors are widely used for biomarker detection, for which these SAW-based biosensor techniques have the potential to transform the cancer and bio-agent detection fields [1]. Most SAW biosensors are composed of a delay-line configuration that consists of interdigital transducers (IDTs) placed on a piezoelectric substrate, with a sensing film between the input and output IDTs. By applying a time-varying electrical signal to the input electrodes, a periodic strain field is generated along the surface, due to the inverse piezoelectric effect, that leads to the propagation of surface waves through the sensing film to be detected on the output IDTs [2]. The principle of SAW sensors is that the mass loading and perturbation on the viscoelastic properties, caused by the attachment of specific molecules to the functionalized sensing film, result in a shift of the frequency or phase that is measured with a frequency counter or network analyzer [3]. Depending on the magnitude of the signal shift, the quantity of the targeted molecules can be determined with a predefined conversion factor. 

In 1979, Wohltjen and coworkers launched the first application of a Rayleigh SAW device for gas detection. However, it has limitations in terms of its application to biosensing in liquid media because of the attenuation of the vertical compression for Rayleigh waves into the surrounding liquid [4]. To avoid the significant damping caused by the liquid environment, it is favorable that the acoustic waves have a shear-horizontal fluctuation which consists of particle displacements parallel to the surface and perpendicular to the direction of the wave propagation. The shear-horizontal SAW (SH-SAW) device, in this regard, has advantages in biological applications because of its low acoustic loss in liquid [5]. SH-SAW sensors have been successfully applied for detecting bacteria, DNA hybrid reaction and cancer cells [6,7,8].

A number of materials have been used for shear-horizontal wave excitation. In particular, 36° Y-X LiTaO_3_ is a piezoelectric material with a large electroacoustic coupling coefficient (*K*^2^) [9]. However, it also has a large temperature coefficient of frequency (TCF), which greatly affects its stability when used for sensors. To make 36° Y-X LiTaO_3_ suitable for an SH-SAW sensor, guiding layers should hence be deposited on the LiTaO_3_ to reduce the TCF of the system. Several materials have been used as the guiding layer in SAW sensors, such as zinc oxide (ZnO) [10], polydimethylsiloxane (PDMS) [11] and silicon dioxide [12]. A guiding layer coated on a LiTaO_3_ substrate can not only decrease the TCF and increase the mass sensitivity but also be functionalized to capture a target for biomolecule detection. Ippolito et al. studied a three-dimensional two-port delay-line SAW device composed of a XY-LiNbO_3_ substrate with a ZnO guiding layer [13]. The piezoelectric displacements and the frequency response of the device were calculated and correlated with experimental data. Trivedi and Nemade have presented a coupled resonance-based SH-SAW delay-line sensor with SiO_2_ micro-ridges [14]. Mass loading was simulated by applying a surface mass density on the SiO_2_ ridges. Their results indicated that the device generates a shear wave with a horizontal displacement component greater than the vertical displacement component. Du et al. proposed a model to quantify the change in the viscoelastic properties of a quartz substrate with a protein layer deposited at the solid/liquid interface [15]. Their model, based on the Sauerbrey equation, provided a useful method for estimating the surface mass coverage of the protein layer. However, the frequency shift of the SH-SAW sensor, corresponding to the concentration of targeted molecules, is difficult to directly extract from this model. 

In this study, two functionalized self-assembly films, 3-aminopropyltriethoxysilane (APTES) and glutaraldehyde, were modified on a SH-SAW device with demonstrated reliability and high sensitivity [16] in order to capture a specific antibody of epidermal growth factor (EGF). EGF has important functions for wound closure and self-repair. Furthermore, as EGF is a key signaling molecule that stimulates epithelial cell motility, it is a factor that is required for re-epithelialization. Increased EGF expression is likely to be a strong portentous and predictive feature for multiple cancer cells [17,18,19]. Therefore, determination of the quantity of EGF has significant benefits for cancer diagnostics. The identification and quantification of EGF are thus particularly important. Standard detection of EGF concentration currently relies on time-resolved immunofluorometric assay (TR-IFMA) [20,21], enzyme-linked immunosorbent assay (ELISA) [22] and fluorescence microscopy [23]. These methods typically are time consuming, complicated and expensive. Recently, label-free biosensors such as electrochemical immunosensors [24] and capacitance sensors [25] have also been reported for detecting EGF in body fluid. To our knowledge, there has not yet been a clear reported development of SH-SAW as an EGF sensor. Therefore, in the current study, we focused on investigating and demonstrating the feasibility of applying SH-SAW for detecting EGF at a concentration that is compatible with biofluids. The sensitivity of these two sensing films was measured and discussed. In addition, most previous numerical studies for SH-SAW devices have focused on the determination of the optimal thickness of the guiding layer to increase the sensitivity [14,26,27]. Few studies investigated the effect on the quantity of frequency shift due to the concentration of sensed biomolecules. In this work, the SH-SAW biosensor was analyzed to obtain the sensing characteristics using numerical simulation. A semi-empirical model was proposed to correlate the concentration of biomolecules with the frequency shift of the SH-SAW sensor. As will be shown in this work, the proposed model, which was modified from the concept of the Sauerbrey equation, provides a useful approach to effectively and accurately analyzing the frequency shift corresponding to the concentration of sensed molecules while greatly reducing the complexity of the mass transport analysis in the sensing film. 

## 2. Materials and Methods

### 2.1. Simulation Method

The electromechanical effect of the piezoelectric SH-SAW device is governed by Equations (1) and (2), which present the surface charge density and mechanical stress relationship for the propagation of acoustic waves in a piezoelectric material under a given electric field:

(1) D=[e][S]+[ϵ]E

(2) $$T=\left[C_{E}\right]\left[S\right]-{\left[e\right]}^{t}E$$

where *D* is the electrical displacement, [*e*] is the piezoelectric constant matrix, [ε] is the dielectric permittivity, *E* is the applied electric field, *T* is the stress tensor, [$C_{E}$] is the elastic constant and [*S*] is the strain tensor. The superscript *t* represents the transpose of the matrix and the ${\left[e\right]}^{t}\;$ matrix is a 3 × 6 matrix [28]. The strain tensor [S] is calculated based on the dielectric constant, the elastic constant, the piezoelectric constant of the material and the applied electric field. These constitutive equations can be solved to relate an applied electrical potential and the induced mechanical displacement. Details of the theory for piezoelectric analysis can be found in [29].

**Figure 1:**
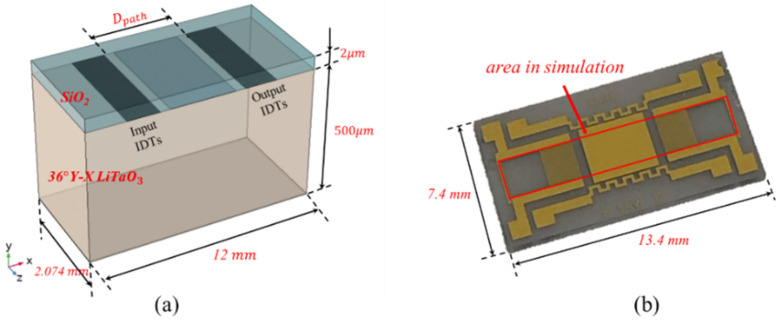
(**a**) Three-dimensional model of the device used in numerical simulation. (**b**) Fabricated SH-SAW chip without SiO_2_ layer. The selected area for simulation is marked with the red rectangle.

COMSOL Multiphysics^®^ was used to solve the SH-SAW characteristics in the current work. Figure 1 shows the geometry of the device simulated in this study. The simulation model was composed of a 36 Y-X LiTaO_3_ substrate and a SiO_2_ guiding layer deposited on the sensing surface. Fifty pairs of IDTs were utilized as transmitting and receiving ports. The sensing area was defined in the middle of these two sets of IDTs. Parameters used in the current IDT’s design are listed in Table 1. This device is designed to have a characteristic wavelength λ_c_ of 34 μm. The substrate thickness is 500 μm, and the thickness of the SiO_2_ guiding layer is 2 μm. The length of the delay-line path length (D_path_) between input and output IDTs is 4420 μm. The width of the structure is 61 λ_c_ in the z-direction in the simulation, which covers the entire length of the IDT fingers on the real device.

For the generation of SAW, an impulse electrical signal of 5 V is applied to the odd electrode fingers of the input IDTs while the even fingers are grounded. The output signals were acquired at the output IDTs. Perfectly matched layers (PML) of 0.1 λ thickness are added to minimize the reflection of the wave from the boundaries of the device [31]. A rotated coordinate system with Euler angles (0°, –54°, 0°) is applied to simulate the desired orientation of the substrate. The mesh consisting of 88,216 elements was decided based on preliminary tests of the grid convergence. 

To investigate the sensitivity of the SH-SAW device, the variation in the resonance frequency with an incremental mass density (Δρ) applied on the surface of the SiO_2_ layer to present the mass loading of the device was calculated by eigenmode analysis. The SAW delay-line device is defined as a dual-port circuit and modeled using an equivalent circuit. The solution in the frequency domain was solved in a range near the designed operating frequency, i.e., 122 MHz based on the SH-SAW velocity and the designed resonance wavelength, as shown in Table 1. The attenuation of the surface acoustic wave during propagation is closely related to the scattering parameter (S-parameter) defined in Equations (3) and (4) [32]:

(3) $$S_{11}=\frac{\left(Y_{0}-Y_{11}\right)\left(Y_{0}+Y_{22}\right)+Y_{12}Y_{21}}{\left(Y_{0}+Y_{11}\right)\left(Y_{0}+Y_{22}\right)-Y_{12}Y_{21}}$$

(4) $$S_{21}=\frac{-2Y_{12}Y_{0}}{\left(Y_{0}+Y_{11}\right)\left(Y_{0}+Y_{22}\right)-Y_{12}Y_{21}}$$

where $Y_{0}$ is the admittance load (typically $Y_{0}$ = 1/50 $\Omega^{-1}$). In the numerical simulation, with voltage signals applied to the terminals, the extracted current is presented as admittance $Y_{ij}$. In the experiment, however, the input and output signals are connected to a vector network analyzer (VNA) that measures the S-parameter as a function of frequency. Equations (3) and (4) state the exact relationships of $Y_{ij}$ and $\;S_{ij}$ and makes it convenient for comparing the numerical and experimental results. 

### 2.2. Fabrication of SH-SAW Sensors

**Figure 2:**
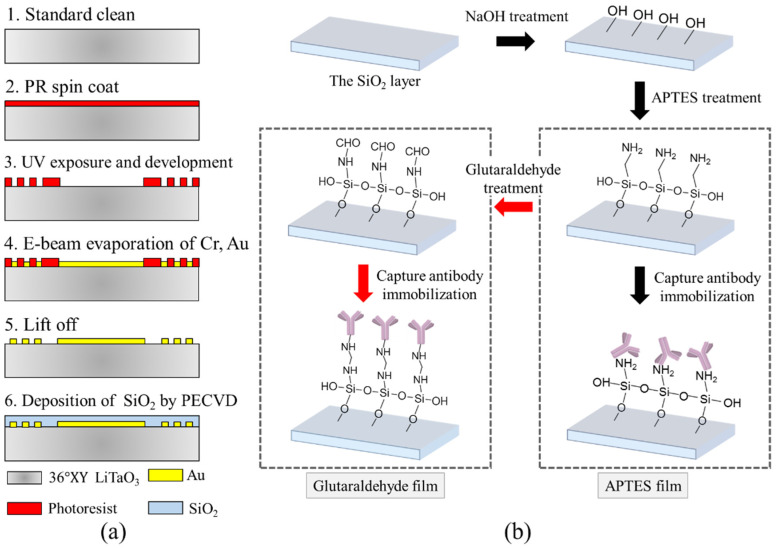
(**a**) Process flow for device fabrication (**b**) Processing steps of biochemical surface modification of the SiO_2_ layer using APTES and glutaraldehyde.

The process flow for fabricating the SH-SAW chip is illustrated in Figure 2a. The IDTs were made using conventional MEMS fabrication techniques with single-mask photolithography. The IDTs consisting of fifty fingers of each port were deposited on the 36° Y-X LiTaO_3_ substrate with an e-beam evaporator. Au/Cr of thickness 100/20 nm, respectively, was deposited, where the Cr layer was used to promote the adhesion of the Au fingers to the substrate. A 2 μm thick SiO_2_ guiding layer was deposited on delay lines and IDTs with plasma-enhanced chemical vapor deposition.

### 2.3. Surface Functionalization

After dicing the wafer, chips were rinsed with standard clean solvent and blown dry with nitrogen. To capture specifically and selectively the target protein, the surface of the sensing section was functionalized. The surface modification process for the SH-SAW chip is illustrated in Figure 2b. The device was first treated with hydroxide by immersing in NaOH (1 M) for an hour. The sensing surface was then silanized by immersing it in a solution of 3-aminopropyltriethoxysilane (APTES, 0.5%, Sigma, A3648) in ethanol (99.5%) for 12 h. After rinsing with pure ethanol and drying with N_2_, the SH-SAW chip was baked in an oven at 95 °C for 2 h to complete the APTES modification of the sensing film.

For the preparation of the glutaraldehyde-modified sensing film, the device was washed with deionized water and dried after the APTES treatment step, and then soaked in a glutaraldehyde solution (5%, Sigma, G7776) diluted in phosphate-buffered saline (pH 7.4, PBS, Sigma, P8313) for 1 h. The chip was then baked in an oven at 95 °C for 2 h to complete the glutaraldehyde sensing film modification. 

To activate the sensing area, capture antibody (anti-EGF, 50 μg/mL) was assembled on the sensing surface of the sensor device for 8 hours, followed by PBS rinse and nitrogen blow drying. When not being used for the experiment, the SH-SAW chip was stored at 4 °C. Both antibody and antigen were obtained from R&D systems, catalog number: DY236. A standard ELISA test was also conducted to confirm the activities of the antibody and antigen.

### 2.4. SH-SAW Sensing System

**Figure 3:**
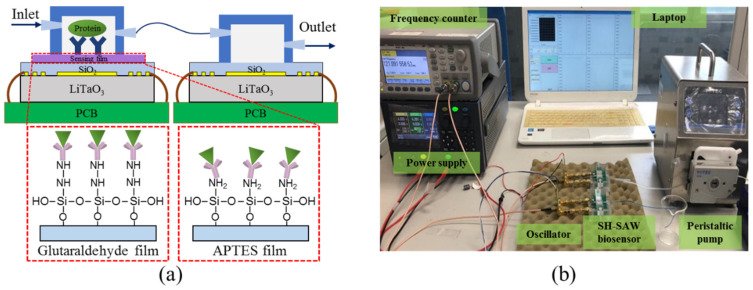
(**a**) Configurations of the dual SH-SAW biosensors and the illustration of EGF antigen binding on these two films. (**b**) Experiment apparatus.

The adsorbent properties of anti-EGF antibody binding on these two sensing films were measured as shown in Figure 3a. A PMMA (Poly(methyl methacrylate) fluidic chamber was designed and fabricated to flow the biomolecule solution on the sensor surface. A dual SAW configuration was used in the experiment to eliminate the noise of signal due to temperature and pressure fluctuations within the environment. Figure 3b is a photo of the experiment setup. A peristaltic pump (YOTEC PS102) was used to ensure a continuous and steady flow of testing fluids in the air-tight system. A positive-feedback circuit was designed to generate and to stimulate the oscillation frequency for signal detection. A power supply (Keysight E3611A) was used to provide the input voltage. The oscillator circuit was connected to the SH-SAW chip to transfer the signal to a frequency counter (Keysight 53220A) and recorded the instant frequency shift using a LabVIEW program.

## 3. Results and Discussion

### 3.1. Numerical Analysis

**Figure 4:**
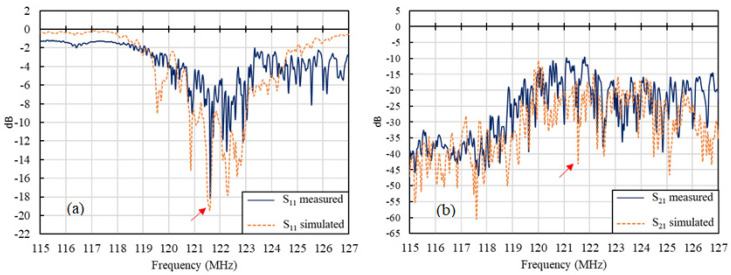
Comparison of (**a**) S11 and (**b**) S21 between simulated and measured frequency responses in the range of major resonance frequencies. Arrows indicate the location of center frequency.

The frequency response for the 36° Y-X LiTaO_3_ SH-SAW sensor was solved numerically, as previously described. Figure 4 shows a comparison of the experimental and simulation results. The S-parameters were measured with a vector network analyzer (VNA). The corresponding values from the simulation were obtained through Equations (3) and (4), as previously noted. As seen in Figure 4a, S_11_ of the simulation results showed the most pronounced resonance frequency at 121.5 MHz, which is very close to that of the measured results (121.6 MHz) and the designed center resonance frequency. The slight deviation on the center frequency from the real device to the designed value can be attributed to the imperfect material properties and fabrication. The insertion loss (IL) S_21_ shown in Figure 4b also suggests good agreement between the simulation and experimental results.

**Figure 5:**
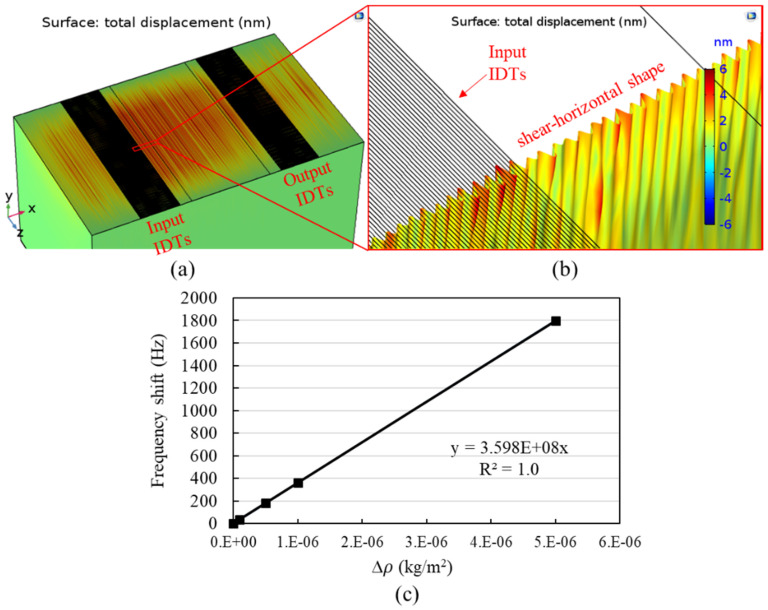
(**a**) The displacement distribution at the center frequency (121.5 MHz) from the eigenmode analysis, (**b**) profile of SH-SAW on a x-y plane along the center line of the device, (**c**) simulated results of frequency shift (Δ*f*) with respect to the incremental surface mass density (Δ*ρ*).

Numerical results of the eigenmode response displacement of the device are shown in Figure 5a. The profile of the acoustic wave in the x-y plane along the centerline of the device in the x-coordinate and near to the input IDTs is shown in Figure 5b. From the piezoelectric displacement, it is obvious that the surface acoustic wave generated is in shear-horizontal shape. For a SAW-based biosensor, sensitivity is an important performance parameter. To attain information regarding the sensitivity, the surface mass density (ρ, kg/m^2^) of the sensing surface was varied in the simulation to determine the corresponding frequency shift. As shown in Figure 5c, for the surface mass density that was varied from 0 to 5 × 10^–6^ kg/m^2^, a nearly linear dependency between the frequency shift (Δ*f*) and the incremental surface mass density Δρ was observed. The slope of the plot represents the mass sensitivity, which is 3.598 × 10^8^ Hz∙m^2^/kg in the current configuration. It should be noted that, to attain the amount of Δρ for a specific SH-SAW sensor corresponding to the concentration of the target molecule, a mass transfer analysis incorporating the surface absorption and adsorption of the molecule on the sensing film is generally required. Here, we proposed an alternative approach to determine the incremental surface mass density with respect to the biomolecule concentration by applying the relationship as follows:

(5) $$\Delta \rho =k_{1}e^{\left(-k_{2}/c0\right)}/m_{sub}$$

where *c*_0_ is the molecule concentration of the measuring sample, *m_sub_* is the mass per area of the substrate, and *k*_1_ and *k*_2_ are empirical coefficients to be determined by comparing the simulation and experimental results. With this proposed model, the dependence of Δ*f* on Δρ can be readily converted to the dependence of Δ*f* on *c*_0_ in the simulation, which is very useful for analyzing and designing SH-SAW biosensors in cases where determining the molecule concentration is crucially important. 

### 3.2. Surface Functionalization

**Figure 6:**
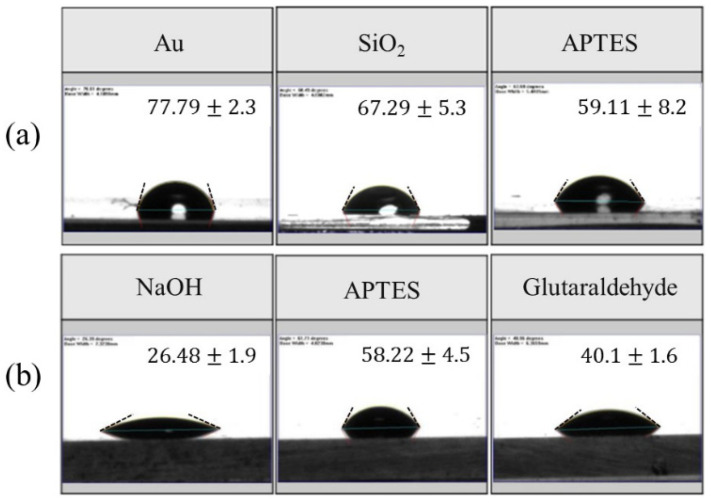
(**a**) Contact angles of Au, SiO_2_ layer and APTES film. (**b**) Contact angles of NaOH treatment, APTES film and glutaraldehyde film. Dash line indicates the asymptotic of the contact line.

Figure 6a shows variation of the water contact angle on the sensing film before and after the APTES modification. The contact angle of the gold layer reduced from 78° to 68° after being coated with a silicon dioxide guiding layer. After it was modified with APTES film, the water contact angle further reduced to 59° and the surface became more hydrophilic. This contact angle is consistent with results reported in the literature [33].

Figure 6b shows the water contact angle variation during the glutaraldehyde film modification. After the NaOH treatment, the SiO_2_ surface was covered with hydrophilic OH groups; thus, the contact angle was reduced to approximately 26.5°. The surface becomes apparently less hydrophilic after the APTES modification. With coated glutaraldehyde polymer, the water contact angle was decreased to 40°, which is more hydrophilic than the APTES modified surface. This result is consistent with the literature [34]. The change in contact angle after every step of surface modification can be considered as an indicator confirming the change in surface properties.

### 3.3. Response Frequency of a SH-SAW Biosensor with EGF Protein

**Figure 7:**
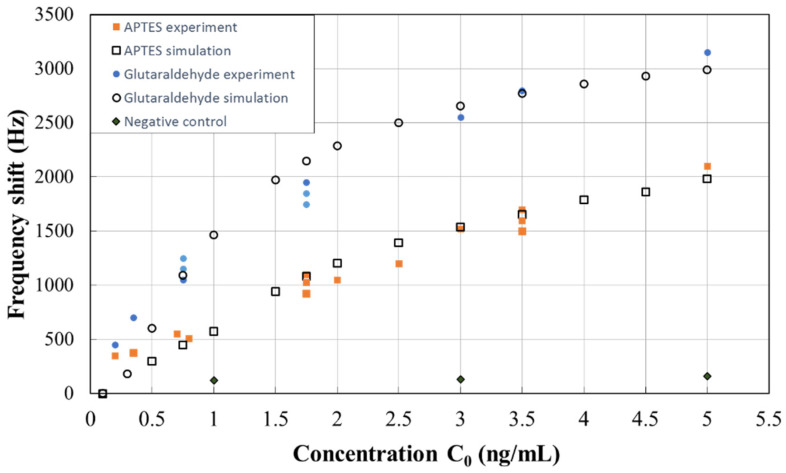
Experimental and simulation results for the frequency shift.

Before the antigen and antibody interaction, BSA solution (1%, w/v) was filled in the microfluidic chamber to achieve an equilibrium initial state for the sensor. The real time frequency signal of the SH-SAW biosensor was measured with samples of EGF concentrations between 0.2 and 5 ng/mL. The stabilized frequency shift varied with the concentration of EGF is shown in Figure 7. According to research in the literature [35], the concentration of EGF in serum and saliva for a normal adult is in the range of 0.5 ng/ml to 3 ng/ml. Therefore, the EGF concentration investigated in this study is in a reasonable range. It should also be noted that a negative control measurement was performed by using an APTES film without anti-EGF antibody. The frequency shift with respect to the EGF concentration from 1 to 5 ng/ml is approximately 100 Hz, as shown in Figure 7. 

It should be pointed out that, as shown in Figure 7, more than ten measurements were conducted with a variety of EGF concentrations on the SH-SAW sensor with each film. Each data point in Figure 7 for the experiments of detecting EGF with APTES and glutaraldehyde films was attained by using a fresh SH-SAW sensor sample to prevent possible contamination. The measurement results showed that both films have clear responses to the concentration of EGF due to mass-loading effects resulting from the antibody–antigen interaction. The slope of frequency shift with respect to the concentration variation of glutaraldehyde film is greater than that of APTES film at a specified EGF concentration. This result suggests that the sensitivity of the chemisorbed film is greater than the physisorbed film. The binding of antibody on the APTES sensing film surface is mainly physical, such as electrostatic and hydrophobic interactions [36], while the glutaraldehyde film forms covalent bonds with the antibody [37], thus ensuring the more stable immobilization of the antibodies. 

### 3.4. Semi-Empirical Correlation Model for a SH-SAW Biosensor

The simulated frequency shift corresponding to the mass loading of the sensing film, as shown in Figure 5c, can be converted to the dependency of frequency shift on EGF concentration by using Equation (5) in conjunction with the experimental results shown in Figure 7. The measurement results of those two sensing films were used to evaluate *k*_1_ and *k*_2_, respectively, and the results are shown in Figure 7 in comparison with the experimental data. Empirical coefficients are determined to be k1:3.7 × 10^–8^, k2:0.89 for the glutaraldehyde film and k1: 2.35 × 10^–8^, k2: 1.48 for the APTES film. It is seen that the proposed model for converting the incremental surface density (Δρ) to the EGF concentration (c_0_) as in Equation (5) yields a good agreement of the simulation results with the experiments. From the semi-empirical model, the sensitivity level is found to be 0.641 and 1.709 kHz/(ng/mL) for APTES and glutaraldehyde sensing films, respectively, at a biomarker concentration of less than 1 ng/mL. Although the estimated frequency shift at low EGF concentrations deviates from the experimental results, the clear response suggests that the SH-SAW sensor and the model are applicable to the analysis of samples with low EGF concentrations. In particular, this model provides an effective simulation model for the frequency response to the EGF concentration without the requirement of solving the detailed transport and adsorption of the biomolecules on the sensing surface. In this way, the response of the SH-SAW sensor to the concentrations of targeted biomolecules can be quickly evaluated from the simulation once those empirical coefficients are determined.

## 4. Conclusions

A SH-SAW biosensor was studied experimentally and numerically to investigate its frequency shift with two types of sensing films, APTES and glutaraldehyde, for detecting cancer-related biomarker antigen EGF, especially at a trace level. For the antigen concentration lower than 1 ng/mL, the frequency shift for the sensor with the APTES film and the glutaraldehyde film is 0.641 and 1.709 kHz/(ng/mL), respectively. Thus, the sensitivity of the glutaraldehyde film to the EGF is higher than that of APTES on the current SH-SAW biosensor. Contact angle measurements of the modified surfaces were conducted to confirm the change in surface properties. Numerical results for the characteristics in resonance frequency are in good agreement with the experimental results. The center frequency was found to be 121.5 MHz and 121.6 MHz from simulation and measurement, respectively. On this basis, a semi-empirical model was successfully developed to correlate the concentration of biomolecules with the frequency shift of the SH-SAW sensor. The proposed model demonstrates a useful approach to effectively and accurately analyze the frequency shift dependency on the concentration of sensed molecules while greatly reducing the complexity of the mass transport analysis in the sensing film.

## Figures and Tables

**Table 1:** IDT parameters.

Parameters	Settings
Designed resonance wavelength (λ_c_)	34 μm
SH-SAW velocity	4100–4212 m/s [30]
Number of fingers	50 pairs
Finger width	8.5 μm
Thickness of electrodes	120 nm
